# The Metacognitions Questionnaire and Its Derivatives in Children and Adolescents: A Systematic Review of Psychometric Properties

**DOI:** 10.3389/fpsyg.2019.01871

**Published:** 2019-09-04

**Authors:** Samuel G. Myers, Stian Solem, Adrian Wells

**Affiliations:** ^1^Division of Psychology, Bar Ilan University, Ramat-Gan, Israel; ^2^Department of Psychology, Norwegian University of Science and Technology, Trondheim, Norway; ^3^Division of Clinical and Health Psychology, The University of Manchester, Manchester, United Kingdom; ^4^Greater Manchester Mental Health NHS Foundation Trust, Prestwich, United Kingdom

**Keywords:** metacognitions questionnaire, children, adolescents, review, psychometrics

## Abstract

**Background:** The Metacognitions Questionnaire (MCQ) and its derivatives have been instrumental in research examining the Self-Regulatory Executive Function Model in adults. Studies testing whether findings are applicable to children and adolescents have been increasing and several different measures adapting the MCQ for younger populations have been developed. The current study aimed to systematically review the psychometric properties of MCQ measures or derivatives used in young people (aged 18 or less), to help assess current findings in this population and to guide future research in this growing area of investigation.

**Method:** Systematic searches were carried out on PubMed and PsycINFO of studies published up to June 2018. Additional studies were identified through Google Scholar and article references. Validity, reliability, range and responsiveness of measures were examined as well as analyses of age and gender differences on scores.

**Results:** Forty-five articles were identified. The total sample consisted of 7,803 children and adolescents (6,922 non-clinical, 881 clinical) aged 7–18. Studies used one of seven versions of the questionnaire, five adapted from the MCQ for younger populations: (1) The Metacognitions Questionnaire-Adolescent version; (2) The Metacognitions Questionnaire-Child version; (3) The Metacognitions Questionnaire-Child Version-Revised; (4) The Metacognitions Questionnaire-Child-30; and (5) The Metacognitions Questionnaire-65 Positive Beliefs Scale Revised; and two adult versions used without adaptation: (1) The Metacognitions Questionnaire-30 and (2) The Cognitive Self Consciousness Scale-Expanded. The validity and reliability of the Metacognitions Questionnaire-Adolescent version had the most extensive support. Other questionnaires had either mixed psychometrics or promising initial findings but more limited data.

**Conclusions:** It is recommended that studies using adolescents (age 12–18) consider using the Metacognitions Questionnaire-Adolescent version. Based on initial data, it is suggested studies using younger populations should consider the Metacognitions Questionnaire-Child-30 but further psychometric research into this and other measures is needed.

## Introduction

### Rationale

The Metacognitions Questionnaire (MCQ-65; Cartwright-Hatton and Wells, 1997) is a 65 item measure that assesses metacognitive belief domains implicated in the Self-Regulatory Executive Function Model of psychological disorder (S-REF; Wells and Matthews, 1994, 1996; Wells, 2000). Metacognition refers to the beliefs, processes and strategies used when cognition is interpreted, monitored or controlled (Flavell, 1979). In the S-REF model, dysfunctional metacognitions lead to perseverative styles of thinking, biased attention, and ineffective self-regulation strategies (the Cognitive Attentional Syndrome; CAS, Wells, 2000) which is considered central to psychological disorder. The MCQ-65 has five subscales assessing the following metacognitions: (1) Positive beliefs about worry (PB), e.g., “Worrying helps me to avoid problems in the future,” (2) Negative beliefs about the uncontrollability and danger of worry (NB), e.g., “My worrying is dangerous for me,” (3) Beliefs about the need for control of thoughts (NFC), e.g., “It is bad to think certain thoughts,” (4) Beliefs concerning cognitive competence (CC), e.g., “I have a poor memory,” and (5) Cognitive self-consciousness (CSC), e.g., “I think a lot about my thoughts.”

To facilitate ease of use, Wells and Cartwright-Hatton (2004) reduced the items of the MCQ and developed the Metacognitions Questionnaire-30 (MCQ-30), a 30 item version of the MCQ with the same factor structure as the original questionnaire, which has become the “gold standard” measure in adult research.

A large number of studies have used the MCQ-65 and MCQ-30 in adult populations. Findings for the MCQ-65 suggest acceptable psychometric properties of the scale (see Wells, 2009 for a review). However, most research has examined the shorter version-the MCQ-30. The five factors of the MCQ-30 have been replicated in several language versions in non-clinical populations (e.g., Spada et al., 2008; Yilmaz et al., 2008; Cho et al., 2012) as well as in populations with psychological disorders (Martín et al., 2014; Grøtte et al., 2016) and physical health difficulties (Cook et al., 2014; Fisher et al., 2016). Although most studies have examined single-order models consisting of the five subscales only, Fergus and Bardeen (2017) examined a bi-factor model consisting of the five subscales, and the total score as a general metacognitive factor, with results supporting this model.

Theoretically consistent positive relationships between MCQ subscales and a range of psychological disorders and symptoms have been shown cross-sectionally (e.g., obsessive-compulsive symptoms, Myers and Wells, 2005; problem drinking, Spada and Wells, 2005; trauma symptoms, Roussis and Wells, 2006; worry, e.g., Wells and Cartwright-Hatton, 2004; psychotic symptoms e.g., Bright et al., 2018) and prospectively (e.g., Sica et al., 2007; Yilmaz et al., 2011). These studies support the trans-diagnostic significance of metacognitive beliefs as proposed by the S-REF model and the convergent validity of the MCQ-30. The negative beliefs about uncontrollability and danger subscale has shown the strongest relationships with symptoms across studies (see e.g., Wells and Cartwright-Hatton, 2004; Spada et al., 2008; Bailey and Wells, 2013) supporting the central nature of this belief in metacognitive theory (see Wells, 2009). Both the MCQ-65 and the MCQ-30 have been shown to differentiate clinical and non-clinical participants, with a meta-analysis looking at both these measures together finding significantly higher scores in a range of clinical groups on all MCQ subscales, with the negative beliefs, and need for control subscales, showing the largest effects (Sun et al., 2017).

Metacognitive Therapy (MCT; Wells, 2000, 2009) is based on the S-REF model and focuses on modifying metacognitive beliefs and strategies. Results from a recent meta-analysis of MCT suggest it is a highly effective therapy for a range of psychological difficulties (Normann and Morina, 2018). Significant changes in the MCQ-30 have been demonstrated following metacognitive treatment (e.g., Wells et al., 2010, 2012). According to S-REF theory, decreases in symptoms following treatments should be mediated by changes in metacognition even when the treatment does not directly target these metacognitions. In support of this, several studies have demonstrated significant changes in MCQ scores following a range of effective non-metacognitive interventions (e.g., Solem et al., 2009; Fernie et al., 2016).

The MCQ has been instrumental in metacognitive research in the adult population. There has been far less research into metacognitive theory and therapy in child or adolescent populations. However, the development of the Metacognitions Questionnaire-Adolescent version (MCQ-A; Cartwright-Hatton et al., 2004) encouraged an increase in metacognitive research in adolescents. The MCQ-A is similar to the MCQ-30 but the wording of some items was modified slightly to make it easier for younger readers to understand. Additionally, the development of versions of the MCQ adapted for children, namely the Metacognitions Questionnaire for Children (MCQ-C30; Gerlach et al., 2008) and the Metacognitions Questionnaire-Child version (MCQ-C; Bacow et al., 2009) has supported metacognitive research in pre-adolescents. These questionnaires were adapted from the MCQ-A by simplifying words and phrases further to make them understandable to a younger age group. Recently the Metacognitions Questionnaire-Child Revised (MCQ-CR; White and Hudson, 2016) has been developed with the aim of making the questionnaire understandable to younger children (from age 7 to 8). Studies using young populations have also used the positive belief scale of the MCQ-65 adapted to be understandable to children (Meiser-Stedman et al., 2007) as well as adult versions of both the MCQ-30 and a measure derived from the cognitive self-consciousness subscale of the MCQ-30, the Cognitive Self Consciousness scale-Expanded (CSC-E; Janeck et al., 2003).

Results using these questionnaires in children and adolescents have been promising, particularly in showing relationships between the MCQ and a range of symptoms (e.g., Cartwright-Hatton et al., 2004; Debbané et al., 2009). A meta-analysis examining the relationships between metacognitive constructs, mainly assessed by MCQ-based measures, and anxiety, found low-medium to high effect sizes for the five factors and total MCQ score (Lønfeldt et al., 2017c). These results appear to support the application of S-REF theory to explaining anxiety and other psychological symptoms in younger populations. However, in assessing this literature it is important to consider the validity of the MCQ measures used in this population. There are several reasons why psychometric findings in adults cannot automatically be assumed to apply to younger populations and adaptations of the scale need to be assessed in children and adolescence populations:

1. Metacognitions develop through childhood and adolescence (Schneider, 2008) and it is not currently known at what age the metacognitions assessed by the MCQ fully develop.
2. The understandability of the MCQ measures to younger participants needs to be assessed.
3. The effects of changes in MCQ measures adapted for younger participants, such as simplifying the language or, with the MCQ-C, removing a subscale, needs to be examined.

It is also important to assess the psychometrics of these questionnaires used with younger populations because the multiple versions of the instrument present a challenge for future researchers in deciding which version to use for which age group of children and/or adolescents. A review and assessment of the psychometrics of these scales would provide information to help inform choices.

### Objective

The aim of the current study was to carry out a systematic review of the psychometric properties of the MCQ and derivatives in children and adolescent populations. It aimed to examine the validity, reliability and responsiveness of the measures. Additionally, it aimed to explore any age or gender differences in scores. Details of the psychometric dimensions assessed in this study are outlined below.

As the central aim of the current study was to evaluate psychometric parameters of the MCQs rather than test theory and because it was possible that the different versions of the scale may have substantive psychometric differences, we did not aim to carry out a meta-analysis of across-measure relationships between metacognitions and symptoms.

#### Validity

Four sources of evidence for validity were examined (see Urbina, 2004; American Educational Research Association, American Psychological Association, & National Council on Measurement in Education, 2014): (1) evidence based on content (2) evidence based on factor structure (3) evidence based on relations with associated measures and (4) evidence based on relations with a criterion.

The current study aimed to assess two aspects of validity evidence related to content: (a) the extent to which the MCQ children or adolescent questionnaires cover the same dimensions of metacognition that the adult MCQ aims to measure, (b) the level of understandability of items to their target population.

Evidence for validity based on factor structure was assessed by examining (a) factor analyses of the measures, (b) whether there was measurement invariance based on gender or age. As the factors of the MCQ are based on theoretically central and distinct forms of metacognition in the S-REF model, and because metacognitions assessed by the MCQ may develop early (see Myers and Wells, 2015), it was hypothesized that the MCQ in children/adolescents would have a similar factorial structure to that found in adults, therefore representing the same set of latent constructs. For these reasons we also hypothesized that the MCQ was likely to be invariant across age, at least in studies that did not include very young children. Based on findings of invariance of the factor structure for men and women in two adult studies of the MCQ (Ramos-Cejudo et al., 2013; Fergus and Bardeen, 2017) we hypothesized that the MCQ in children/adolescents would also be invariant across gender.

Consistent with S-REF theory, the MCQ-30 has been shown to positively and significantly correlate with a range of symptoms in adults. The current study aimed to assess evidence of validity of the questionnaires in children and adolescents by examining the size and significance of correlations between the MCQ total score and subscales and validated symptom measures. Based on previous findings in adults, described earlier, we hypothesized that of the subscales, negative beliefs about uncontrollability and danger (NB) would have the strongest and most consistent relationships across symptom dimensions, with the other subscales also showing relationships but of a more specific nature and of lower magnitude.

One form of validity evidence based on relations with a criterion, is the ability to detect group differences (see Cronbach and Meehl, 1955). It was hypothesized that MCQ scores would be significantly higher in clinical than non-clinical groups. Results in adults (see meta-analyses by Sun et al., 2017) suggest these should exist for most subscales and across disorders but that the most consistent and strongest differences should be for NB and Need for Control (NFC) with moderate effects for Cognitive Confidence (CC) and Cognitive Self-Consciousness (CSC) and less strong and reliable effects for Positive Beliefs (PB).

#### Reliability

Two forms of reliability were tested: (a) the internal consistency of the subscales and of the total score, (b) test-retest reliability, as a test of the stability of the measure over time.

#### Distribution of Scores

We also examined whether the total score and subscales of the MCQ measures presented a range of scores, as a restricted range would impact on both validity and reliability of the measures.

#### Responsiveness

Responsiveness refers to the ability of a measure to detect changes in the construct being measured. We aimed to assess whether the MCQ measures in children and adolescents changed following successful treatment. It was hypothesized that there would be some change on MCQ scores following any form of treatment which led to symptom changes but that decreases in MCQ scores would be particularly apparent following Metacognitive Therapy which directly targets metacognitions.

#### Age Differences

Our study aimed to explore the presence of any age differences in the metacognitions measured by the MCQ within this population. General metacognitive skills and knowledge first develop in childhood (e.g., Schneider, 2008). Implicit metacognitions may already be present in 2 month old infants and some children as young as three can report on their metacognitions to some extent (Marulis et al., 2016), with a significant quantitative and qualitative increase in metacognitive skills between age 5 and 7 (Bryce and Whitebread, 2012). Metacognitive knowledge and some metacognitive skills continue to develop through adolescence (Schneider, 2008). Thus, the detection of metacognitions measured by the MCQ may vary depending on age.

#### Differences Between Sexes

The study also aimed to examine if there were any differences between sexes in MCQ scores. Studies in adults have produced somewhat inconsistent results with some studies finding no differences (Wells and Cartwright-Hatton, 2004; Grøtte et al., 2016) and others finding differences in some individual subscales (Spada et al., 2008; O'Carroll and Fisher, 2013). Fergus and Bardeen (2017) suggest that this inconsistency may be explained by the fact that any differences between sexes in MCQ scores that exist may be small. It was therefore hypothesized that there would be no consistent differences between sexes in scores on the MCQ in children and adolescents.

## Methods

### Eligibility Criteria

Eligibility criteria for inclusion were:

1. Articles written in English, published or in press in a peer reviewed journal up to June 2018.
2. Participants or a reported sub-sample were all 18 years of age or under.
3. The MCQ or subscales or a questionnaire explicitly derived from the MCQ or subscales was used.

Articles were excluded if they had an English abstract but the main text was not in English or they analyzed results for participants aged 18 (or younger) together with older participants.

### Search Strategy

Searches were carried out on PubMed and PsycINFO, using Boolean logic and the following keywords:

- Child OR adolescent OR adolescence

AND

- Metacognitions Questionnaire OR Meta-cognitions Questionnaire:

Additional searches were carried out on Google Scholar. References in identified articles were also examined for relevant articles.

### Data Extraction

The following information was extracted from all articles where present:

1. Details about sample namely: size, clinical/non-clinical status, age range, and mean age.
2. Country where research was carried out.
3. Metacognition questionnaire and symptoms questionnaires used.
4. The Reading-Grade Level of the measure and data on the measures' understandability to participants.
5. Factor analysis results: number and types of factors found and results of fit indices, tests of measurement invariance.
6. Internal consistency, as measured by Cronbach alphas, for the subscale and total score.
7. Test-retest reliability: time period measured, interclass correlational results.
8. Ranges of scores.
9. Results of correlations between MCQ measures and validated symptom measures.
10. Comparisons of MCQ measure scores between clinical and non-clinical samples.
11. Analysis of age, gender or age by gender differences in MCQ measure scores.
12. The effects of interventions or treatments on MCQ scores.

Where studies used the same or overlapping samples as previous studies, the results were only extracted when these were separate analyses to those reported previously.

Factor analysis results of both exploratory and confirmatory factor analysis were examined. All absolute and comparative fit indices reported were extracted apart from Chi-square because of its sensitivity to sample size (Bentler and Bonett, 1980). Studies reported one or more of the following fit indices: Absolute fit indices: Goodness of Fit Index (GFI); Adjusted Goodness of Fit Index (AGFI); Root Mean Square Error of Approximation (RMSEA); Root Mean Square Residual (RMSR). Comparative Fit indices: Normed Fit Indices (NFI); Relative Fit Index (RFI); Comparative Fit Index (CFI); Parsominous Fit Index (PFI). The following criteria were used to assess these fit indices. For the RMSEA 0.08 and less was considered adequate and 0.05 and less was considered good (see MacCallum et al., 1996). For the RMSR less than 0.08 is considered good (Hu and Bentler, 1999). For all other indexes 0.90 was considered adequate and 0.95 good (see Bentler and Bonett, 1980; Hu and Bentler, 1999;Kline, 2005).

When assessing Cronbach alpha scores and test-retest interclass correlations we used the guidelines given by the bib27 Review Model 2013: Cronbach alphas *r* < 0.70 = Inadequate 0.70 ≤ *r* < 0.80 = Adequate, 0.80 ≤ *r* < 0.90 = Good, *r* ≥ 0.90 = Excellent; Test-retest *r* < 0.60 = Inadequate, 0.60 ≤ *r* < 0.70 = Adequate, 0.70 ≤ *r* < 0.80 = Good, *r* ≥ 0.80 = Excellent.

For tests of validity based on associated measures, we included any correlations reported between symptom measures which were based on child-report and had been validated in at least one prior study, and the MCQ measures. We did not include the few correlations reported between an MCQ measure and a measure of child symptoms as reported by parents as evidence suggests significant disparity between child and parent reports of symptoms (De Los Reyes and Kazdin, 2005; Canavera et al., 2009). As concurrent validity rather than specificity was being tested, we did not include correlations or regressions that controlled for other symptoms e.g., correlations between the MCQ and anxiety controlling for worry.

For tests of validity based on relations with a criterion, where differences between clinical and non-clinical groups were reported as significant we calculated effect sizes based on the means, standard deviations and number of participants, using RevMan Software.

The assessment of effect sizes was based on Cohen (1988), for correlations (*r*), 0.1 = small, 0.3 = medium and 0.5 = large; for differences between means, Cohen's *d*, 0.2 = small 0.5 = medium and 0.8 = large.

When assessing the effects of treatments or interventions on MCQ scores we also examined the effectiveness of the intervention on primary outcome measures, as decreases in metacognition would only be expected following a successful intervention.

Results of psychometrics are presented based on the suggested order of evaluating measurement properties suggested by the COSMIN methodology (Prinsen et al., 2018). Validity evidence based on content was assessed first as initially it is important to assess whether a measure is comprehensive and comprehensible (Prinsen et al., 2018). Then the internal structure was examined by assessing validity based on factor structure and internal consistency. Then other reliability and validity evidence were assessed followed by responsiveness. Age and gender analyses of differences in scores were exploratory and were examined last.

### Quality Assessment of Studies

The methodological quality of studies was assessed on the following criteria, based on a modified version of the Newcastle-Ottowa scale for cross-sectional studies (Herzog et al., 2013): (1) Research question and design; (2) Sampling method; (3) Sample size; (4) Data collection; (5) Method of dealing with missing data; (6) Analysis (Appendix with scoring system). The maximum possible score if all criteria were met was 8. Two of the authors independently marked the studies and any differences in scores were discussed and resolved.

## Results

### Search Results and Study Characteristics

**Figure 1:**
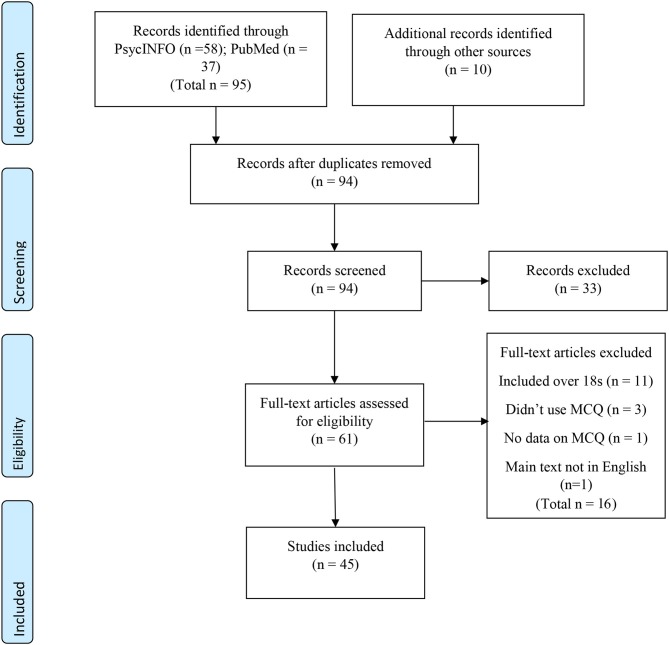
PRISMA diagram of search and study selection process.

A PRISMA diagram (Moher et al., 2009) of the search results and study selection process is presented in Figure 1.

As shown, 105 articles were produced by the literature searches. Of these, eleven were duplicates. Ninety-four articles were screened, 33 were rejected at the screening stage as examination of title and/or abstract showed they either clearly included participants over the age of 18 or were not peer-reviewed articles. Of the remaining 61, 16 were excluded as examination of the full text showed they either: (1) included participants over 18, (2) did not use an MCQ measure, (3) did not have data on the MCQ or (4) were not in English. Forty-five articles met the inclusion criteria and were included in the review. These articles consisted of 34 separate groups of participants. Descriptions of the methodologies, a score for the quality of the studies as well as a summary of psychometric and other findings for the 45 articles are shown in Table 1.

**Table 1:** Study methodology, quality score, psychometrics and main findings relevant to the review.

**Study and quality score**	**Participants, country study was carried out in and design**	**MCQ measure and symptom measures included in review**	**Factor analysis, reliability and ranges of scores**	**Findings relevant to concurrent and criterion validity and age/gender analyses**
Cartwright-Hatton et al. (2004) Quality Score = 4	*N =* 166 non-clinical and 11 clinical “an emotional disorder” Age 13–17 Mean 15.3 UK Design: Cross-sectional	MCQ-A total score and subscales RCMAS CDI LOI-CV	Factor analysis: Principal components factor analysis showed a similar five factor structure to the adult version—MCQ-30 Internal consistency for total scale and subscales adequate to good (76–0.86) with the exception of NFC (0.66) Test-retest reliability over two weeks good to excellent for all subscales (0.77 to 0.90) apart from negative beliefs about worry (0.24) and total score (0.34) Flesch-Kincaid Reading Grade Level of 3.6	All subscales and the total score significantly and positively correlated with measures of anxiety, depression, and obsessive-compulsive (o-c) symptoms A comparison of the clinical sample with a paired (by gender and age) subset of the non-clinical sample showed that the clinical group scored significantly higher on three subscales—NB; NFC, and CC as well as on MCQ-A -total score but not on positive beliefs or cognitive self-consciousness No gender differences on any MCQ-A scores
Mather and Cartwright-Hatton (2004) Quality Score = 5	Same non-clinical sample as above Design: Cross-sectional	MCQ-A total score LOI-CV CDI	Range of MCQ Total Score 33–116	
Matthews et al. (2007) Quality Score = 6	*N =* 223 non-clinical Age 13–16 UK Design: Cross-sectional	MCQ-A total score and subscales LOI-CV no. and interference	Internal consistency adequate to excellent for all subscales and total score (0.75–0.91) Range: MCQ-Total 30–102; PB 6–22; NB 6–24; CC 6–22; NFC 6–20; CSC 6–23	MCQ-A Total and subscales significantly and positively correlated with both number of o-c symptoms and level of interference from them No gender differences on any measures MCQ-Total, NB, NFC and CSC significantly negatively correlated with age in months, although correlations were low −16 to −19
Debbané et al. (2009) Quality Score = 5	*N =* 81 non-clinicals and 82 from psychiatric outpatient service Age 12–18 Mean 15.3 Switzerland Design: Cross-sectional	MCQ-A total score and subscales SPQ	Range: MCQ-Total 35–108	With age and IQ controlled MCQ-A total score and all subscales apart from CSC significantly and positively correlated with positive schizotypy range 0.31–0.57 in both the total sample and a subsample with hallucination symptoms
Crye et al. (2010) Quality Score = 5	*N =* 62 non-clinical Age 12–14 Mean 13 years 4 months (SD 0.67) UK Design: Cross-sectional	MCQ-A total score LOI-CV	–	The MCQ-A total score had a positive and significant correlation with the LOI-CV No gender differences on any variables
Wilson et al. (2011) Quality Score = 4	*N =* 72 non-clinical (part of parents and children dyads) Age 11–16 Mean 13.2 (1.04) UK Design: Cross-sectional	MCQ-A total score and subscales MASC PSWQ-C	Internal consistency adequate to good for subscales and total scores 0.76–0.86 with the exception of NFC = 0.57	MCQ-A subscales apart from CC significantly correlated with worry (MCQ-total not included) Only UCD significantly correlated with anxiety. No age or gender differences
Ellis and Hudson (2011) Quality Score = 6	*N =* 123 42 non-clinical Age: 12–17 Mean 13.7 (1.4) 81 clinical sample Age: 12–17 Mean 14.1 (1.5) 35 boys 46 girls Australia Design: Cross-sectional	MCQ-A totals score and subscales	Factor analysis: Good or adequate fit on most fit indices in Confirmatory Factor Analysis Internal consistency adequate to excellent for subscales and total score 0.77–0.92	PB, UD, NFC, and Total Score significantly higher in Clinical vs. Non-Clinical group No age correlations or gender differences
Wolters et al. (2012) Quality Score = 5	*N =* 317 non-clinical and 40 OCD clinical sample Age 12–18 Mean 14.1 Holland Design: Cross-sectional	MCQ-A Dutch version total score and subscales LOI-CV RCADS	Confirmatory factor analysis showed adequate fit with most fit indices above or equal to 0.9 for a five-factor model both with and without a higher order factor (total score). Best fitting model had three items removed but was not used as model found in previous studies was adequate Internal consistency of total scale and subscales adequate to excellent in both non-clinical and clinical samples (0.70–0.92) with the exception of NFC in the non-clinical sample (0.65). Test-retest reliability over 6 to 21 weeks in non-clinical and clinical population good to excellent for all subscales and total score (0.72 to 0.95) apart from NFC in non-clinical sample (0.35) Ranges for clinical: Total 36–104, PB 6–22, NB 6–24, CC 6–22, NFC 6–22, CSC 7–24 non-clinical: Total 30–96, PB 6–24, NB 6–21, CC 6–21, NFC 6–19, CSC 6–24	Significant correlations with several anxiety subscales and depression in non-clinical and clinical samples for PB, UD, and CC. NFC and CSC in general only significantly related to anxiety and depression measures in non-clinical (and larger) sample A comparison of the clinical sample with the non-clinical sample showed that the clinical group scored significantly higher on the MCQ-A total score as well as all subscales apart from CC In non-clinical sample, small positive relationship between MCQ-A total scale and age *r* = 0.12. No relationship in clinical sample
Wilson and Hall (2012)Quality Score = 5	*N =* 151 non-clinical Age 13–16 Mean 15.05 (1.03) UK Design: Cross-sectional	MCQ-A total score and subscalesLOI-CV	–	All MCQ-A subscales and the total score significantly and positively correlated with obsessional symptoms apart from CSC
Farrell et al. (2012) Quality Score = 3	*N =* 46 clinical (all with OCD diagnosis) Age 24 participants 7–11 and 22 participants 12–17 Mean age 11.3 (2.86) Australia Design: Cross-sectional	MCQ-A total score CY-BOCS	Internal consistency for MCQ-A total score was good in children (0.87) and excellent in adolescents (0.92)	MCQ-A total score significantly correlated with o-c symptom severity in the adolescent but not the child sample
Mazloom et al. (2016) Quality Score = 6	*N =* 678 non-clinical Age 14–18 Mean age 15.81 Azerbaijan Design: Cross-sectional	MCQ-A total score (Farsi version) PSS-SR	Internal consistency for MCQ-A total score was good (0.84)	MCQ-A Total score significantly correlated with post-traumatic symptom severity
Sanger and Dorjee (2016) Quality Score = 5	*N =* 38 non-clinical Age 16–18 UK Design: Pre-post	MCQ-A	–	A Mindfulness intervention led to significant reductions in MCQ-A total score and NFC as compared to a control group Pre-post differences on NB were correlated with changes in N2
Bacow et al. (2009) Quality Score = 2	*N =* 78 clinical 20 non-clinical Age 7–17 Mean age: clinical group 11.86 (3.11) non-clinical group 12.41 (3.02) USA Design: Cross-sectional	MCQ-C PSWQ-C CDI ADIS-IV C/P GAD section	Confirmatory factor analysis reported as adequate fit but fit indices suggest poor to adequate fit Internal consistency for total scale and subscales adequate to good (0.75–0.87) with the exception of NFC (0.64) Flesch-Kincaid Reading Grade Level of two	Total sample MCQ-C and subscales significantly correlated with worry. NB, CSC, and total score significantly correlated with depression With worry content as covariate only significant difference between clinical and non-clinical group on subscales was significantly higher levels of CSC in non-clinical group Positive relationship between CSC and age (examined in clinical sample only) but only unstandardized regression coefficient brought (0.46) Interaction between age and gender on MCQ-subscales or total score was not significant for younger participants (1 SD below mean age). However, in adolescents (1 SD above mean age) girls scored higher than boys on the MCQ-C total score only
Bacow et al. (2010) Quality Score = 4	Same sample as above	MCQ-C ADIS-IV C/P GAD section		With worry content as covariate and different anxiety groups as well as a non-clinical group compared, only significant difference was higher levels of CSC in non-clinical group compared to Separation Anxiety Disorder group
Irak (2012) Quality Score = 6	*N =* 470 non-clinical Age 8–17 Mean 12.2 (2.8) Turkey Design: Cross-sectional	MCQ-C (Turkish version) STAI-C MOCI (All Turkish versions)	Confirmatory factor analysis indices suggest adequate fit Internal Consistency adequate (0.73) Test-retest Good to excellent (Range 0.76–0.82)	Significant correlation between MCQ-C total and subscales and trait anxiety and o-c symptoms. Age significant only for PB higher in older group Females scored higher than males on NB and total score. No age/gender interaction
Boysan et al. (2016) Quality Score = 5	*N =* 805 non-clinical Age 11–17 Mean 13.85 (1.4) Turkey Design: Cross-sectional	MCQ-C (Turkish version) LOI-CV	–	MCQ-C total score and subscales significantly correlated with total score and subscales of o-c symptoms
Benedetto et al. (2014) Quality Score = 5	*N =* 184 non-clinical Age 11–13 Mean 11.96 (0.9) Italy Design: Cross-sectional	MCQ-C (Italian version) PSWQ-C RCMAS-2	Internal consistency for subscales inadequate to good (0.61–0.78)	Significant correlation between MCQ-C subscales and worry as well as trait anxiety No gender differences
Kertz and Woodruff-Borden (2013) Quality Score = 4	*N =* 80 non-clinical Age 8–12 Mean 9.6 (1.1) USA Design: Cross-sectional	MCQ-C PB and NB scales PSWQ-C RCMAS worry/oversensitivity subscale SPSI (subscale)	–	NB but not PB significantly correlated with anxiety PB distinguished between clinical and non-clinical worriers based on a clinical cut off score
Smith and Hudson (2013) Quality Score = 4	*N =* 83, 49 clinical (anxiety disorders), 34 non-clinical Age: 7–12 Mean 9.18 (1.56) USA Design: Cross-sectional	MCQ-C SCAS SDQ ADIS IV C/P	Internal consistency adequate for total score (0.73), subscales inadequate (0.25 to 0.64)	MCQ-C total score and some subscales correlated with SCAS and SDQ_E Anxiety group had significantly higher scores than controls on MCQ-C total score PB and NB Examination of understanding of MCQ-C
Fisak et al. (2014) Quality Score = 5	*N =* 175 non-clinical Age: 11–18 Mean 13.94 (1.52) USA Design: Cross-sectional	MCQ-C PSWQ-C	Internal consistency adequate for PB (0.74) other subscales inadequate (0.56 to 0.64)	
Holmes et al. (2014) Quality Score = 7	*N =* 42 clinical GAD patients Age: 7–12 Mean 9.64 (1.41) Australia Design: pre-post scores	MCQ-C	Internal consistency PB and NB adequate (0.78 and 0.76)	NB significantly lower at 3 months in both WLC and treatment groups no change on PB
Donovan et al. (2016) Quality Score = 5	*N =* 25 clinical GAD patients and 25 non-clinicals Age: 7–12 Mean Australia Design Cross-sectional	MCQ-C	Overlapping samples	Significant difference between GAD and non-clinical group on NB but not PB
Donovan et al. (2017) Quality Score = 5	*N =* 114 non-clinicals Age: 8–12 Mean 9.87 (1.30) Australia Design Cross-sectional	MCQ-C PSWQ-C	Internal Consistency PB 0.54 (inadequate) NB 0.72 (adequate)	PB and NB correlated with worry
Kadak et al. (2013) Quality Score = 5	*N* = 738 non-clinical exposed to earthquake Age: 13–17 Mean 16.22 (0.88) Turkey Design: Cross-sectional	MCQ-C T CPTDS-R I STAI-C SCARED-R-CV CDI CASI A-DES	–	MCQ-C T correlated with dissociation, anxiety and depression
Carr and Szabó (2015) Quality Score = 5	*N =* 93 non-clinical Age: 7–12 Mean 10.0 (1.19) Australia Design: Cross-sectional	MCQ-C PB CAWS-Worry	PB Internal consistency (0.69)	No relationship between age and PB scores and no gender differences
Stevanovic et al. (2016) Quality Score = 5	*N =* 258 non-clinical 209 clinical Age: Non-clinical 12–15 Mean 13.09 (0.79) Clinical 9–18 Mean 13.96 (2.29) Design: Cross-sectional	MCQ-C (Serbian Version)	Exploratory and Confirmatory factor analysis EFA showed poor fit of 4 factor model, 3 factor model had good fit and 3 items of this model were removed after CFA	
Hearn et al. (2017a) Quality Score = 6	*N =* 126 clinical Social Anxiety Disorder (SAD) Treatment 95 WL 30 Age: 8–17 Mean 11.29 (2.67) Australia Design: Cross-sectional	MCQ-C PB and NB scales PSWQ-C-SF SPAI-C/P	Internal Consistency PB 0.75 (adequate) NB 0.65	NB but not PB correlated with symptoms
Hearn et al. (2017b) Quality Score = 7	GAD sample but not SAD sample overlapping	MCQ-C	Overlapping samples	SAD group scored higher than non-clinical group on some MCQ-C measures
Hearn et al. (2018) Quality score = 7	*N =* 125 clinical SAD Treatment 95 WL 30 Age: 8–17 Mean 11.28 (2.68) Australia Design: correlation and pre-post	MCQ-C PB and NB scales PSWQ-C-SF SPAI-C/P	Overlapping samples	Significant reductions reported for PB and NW only at 6 months follow-up
Francis et al. (2017) Quality Score = 6	*N =* 312 non-clinical Age: 9–15 Mean 11.9 (1.23) Australia Design: cross-sectional	MCQ-C	Internal Consistency Total Score good 0.86	
Francis et al. (2018) Quality Score = 6	*N =* 312 non-clinical Same sample as above Age: 9–15 Mean 11.9 (1.23) Australia Design: Cross-sectional	MCQ-C PSWQ-C	Internal Consistency PB 0.85 (good), NB 0.78 (adequate) Ranges: PB 6-22 NB 6-24	Significant correlations between PB and MB and PSWQ-C
Esbjørn et al. (2013) Quality Score = 7	*N =* 974 non-clinical Age 9–17 Mean: not reported Denmark Design: Cross-sectional	MCQ-C30 Danish PSWQ-C SCARED-R	Confirmatory factor analysis showed adequate fit for a five-factor model with a higher order factor (total score) Internal consistency of total scale and subscales adequate to good (0.75–0.87) with the exception of NFC (0.6)	Significant correlation between MCQ-C subscales and total score and worry as well as trait anxiety Gender correlation differences mediated by anxiety No significant age differences in model fit indices for measurement model or structural model including anxiety symptoms
Esbjørn et al. (2015) Quality Score = 6	Study 1 *N =* 587 sample of non-clinical sample above Age 9–17 Mean 12.59 (1.66) Study 2 *N =* 93 (new sample) 22 Generalized Anxiety Disorder (GAD) patients, 28 Anxiety Disorder (AD), 43 Non-Clinical Age 7–12 Mean 9.78 (1.64) Denmark Design: Cross-sectional	MCQ-C30 Danish PSWQ-C SCARED-R ADIS-IV-CP	Internal consistency for sample aged 7-8 Total score 0.91 (excellent), PB 0.73, NB 0.71, CSC 0.75 (adequate), NFC 0.62, CC 0.69	GAD group had significantly higher scores than controls on all MCQ-C30 subscales apart from CSC GAD group had significantly higher scores than AD group on NB AD group had significantly higher scores than control group on NB and NFC
Normann et al. (2016) Quality Score = 6	*N =* 44 clinical pre and post treatment and 39 follow-up; sample related to clinical sample above Age 7–12 Mean 9.86 (1.64) Denmark Design: pre-post	MCQ-C30 Danish SCARED-R	Overlapping sample	MCQ-C30 Total score significantly reduced following CBT treatment at post treatment and reduced further significantly from post-treatment to follow-up Changes in MCQ-C30 T significantly related to changes in anxiety at post-treatment but not follow-up
Esbjørn et al. (2016). Quality Score = 5	*N =* 111 non-clinical Age: 8–12 Mean 9.18 (1.56) Denmark Design: Cross-sectional	MCQ-C30 Danish RCADS anxiety PSWQ-C	Internal consistency good for total score (0.89) and CC (0.82), NB (0.78), and CSC (0.73) adequate, PB (0.64) and NFC (0.59) inadequate	MCQ-C total score significantly correlated with anxiety and worry symptoms total scores
Lønfeldt et al. (2017b) Quality Score = 7	*N =* 1062 Non-Clinical related to Esbjørn et al. (2013) Age 9–17 Mean 12.26 (1.25) Denmark Design: Cross-sectional	MCQ-C30	Overlapping sample	Older age significantly related to lower MCQ total score (−0.08)
Lønfeldt et al. (2017a) Quality Score = 6	*N =* 188 Non-Clinical sample related to Esbjørn et al. (2016) Age 7–12 Mean 10.01 (1.41) Denmark Design: Cross-sectional	MCQ-C30	Overlapping sample	NB significantly higher in girls than boys. No other gender differences
Esbjørn et al. (2018) Quality Score = 5	*N =* 44 Age: 7–13 Mean 9.68 (1.60) Denmark Design: pre-post	MCQ-C30 Danish	Internal Consistency 0.86 to 0.87 (good) for total score across 3 timepoints	MCQ-C total score and most subscales significantly changed pre to post treatment
Campbell et al. (2018) Quality Score = 5	*N =* 23 High functioning ASD Ages 8–12 Australia Mean 10.38 (1.39) Design: Cross-sectional	MCQ-C30 English RCADS	Internal consistency: Total Score 0.69, PB 0.87, NB 0.65, CC 0.66, NFC 0.68, CSC 0.62	NB, NFC and Total Score significantly correlated with RCADS anxiety and depression total score
White and Hudson (2016) Quality Score = 5	*N =* 187 non-clinical Age: 7–12 Mean 10.57 (1.69) Australia Design: Cross-sectional	MCQ-CR SCAS PSWQ-C	Factor analysis: Confirmatory Factor Analysis showed different acceptability of 5 factor structure depending on which test of fitness Internal consistency: adequate to excellent for subscales and total score (0.76 to 0.90). Ranges Total score 30-104, PB 6-22, NB 6–24, CC 6–23, NFC 6–24, CSC 6–23.	MCQ-CR total score and subscales significantly correlated with anxiety and worry symptoms Negative correlation between age and CSC (−0.36) and NFC (−0.15) Negative correlation between age and understanding of items −0.23 Examination of understandability No significant difference between males and females
Jacobi et al. (2006) Quality Score = 4	*N =* 126 non-clinical Age: 15–17 Mean 16.2 (1.2) USA Design: Cross-sectional	Cognitive self-consciousness Scale-Expanded (CSC-E) Padua Inventory	Internal consistency adequate (0.77)	CSC-E significantly correlated with o-c symptoms
Gallagher and Cartwright-Hatton (2008) Quality Score = 4	*N =* 168 non-clinical Age 16–18 Mean 17.23 (0.86) UK Design: Cross-sectional	MCQ-30 total score STAI-T	–	MCQ-30 total score significantly correlated with anxiety
Welsh et al. (2014) Quality Score = 3	*N =* 31 at risk of psychosis and 76 non-clinical Age 12–18 Mean 17.23 (0.86) UK Design: Cross-sectional	MCQ-30	-	Group at risk of psychosis scored significantly higher on NB, CC, NFC, and MCQ-T than controls
Meiser-Stedman et al. (2007) Quality Score = 5	*N =* 93 children subjected to assault or MVA Age 10–16 Mean 13.9 (1.9) UK Design: Cross-sectional	The Metacognitions Questionnaire-65 Positive Beliefs scale Revised (MCQ-PBR) RIES-C	Internal consistency excellent (0.9)	MCQ-PBR significantly correlated with trauma symptoms, as well as Acute Stress Disorder (ASD) but not “early Post Traumatic Stress Diagnosis (PTSD)” Participants who met criteria for ASD had significantly higher MCQ-PBR scores than those who did not. However, there was no significant difference on MCQ-PBR scores between participants with “early PTSD” and those without
Meiser-Stedman et al. (2009) Quality Score = 5	*N =* 59 of above sample Mean age 14 (1.8) Design: Prospective	As above		Time 1 MCQ-PBR significantly correlated with six-month trauma symptoms but controlling for Time 1 trauma symptoms removed the significance of this relationship Time 1 MCQ-PBR did not differentiate between those who did and did not meet PTSD criteria six months after a trauma

*ADIS-IV-C/P, Anxiety Disorders Interview Schedule-Child/Parents Version; CAWS-Worry, Child and Adolescent Worry Scale; CDI, Children's Depression Inventory-Short Form; CY-BOCS, Children's Yale-Brown Obsessive-Compulsive Scale; LOI-CV, Leyton Obsessional Inventory–Child Version; MASC, Multidimensional Anxiety Scale for Children; MCQ, Metacognitions Questionnaire, -A Adolescent version, -C Child Version, -C30 Child-30; MCQ Subscales; PB, Positive beliefs about worry; NB, Negative beliefs about worry; CC, Cognitive Confidence; NFC, Need for control; CSC, Cognitive self-consciousness; CR, Child Revised; MOCI, Maudsley Obsessive-Compulsive Inventory; Penn State Worry Questionnaire for Children (PSWQ-C); PSS-SR, Post Traumatic Stress Disorder Symptom Scale Self-Report; RCADS, Revised Children's Anxiety and Depression Scale; RCMAS, Revised Children's Manifest Anxiety Scale; SCARED, Screen for Child Anxiety Related Disorders -r: Revised; SCAS, Spence Children's Anxiety Scale; SPQ, Schizotypal Personality Questionnaire (SPQ); SDQ, Strength and Difficulties Questionnaire; SPAI, Social Phobia and Anxiety Inventory; STAI, State Trait Anxiety Inventory, -C Child version, -T Trait version; RIES-C, Revised Impact of Event Scale-Child Version*.

In total there were at least 7,803 separate participants in the studies. Of these 6,922 were non-clinical and 881 were clinical. Ages ranged from 7 to 18.

### Metacognitions Questionnaires Used

One of seven versions of the Metacognitions Questionnaire or a subscale or subscales of the MCQ were used:

1. The Metacognitions Questionnaire-Adolescent version (MCQ-A) (Cartwright-Hatton et al., 2004).
2. The Metacognitions Questionnaire-Child version (MCQ-C; Bacow et al., 2009).
3. The Metacognitions Questionnaire-Child Version-Revised (MCQ-CR; White and Hudson, 2016).
4. The Metacognitions Questionnaire-Child-30 (MCQ-C30; Gerlach et al., 2008).
5. The Metacognitions Questionnaire-30 (MCQ-30; Wells and Cartwright-Hatton, 2004).
6. The Metacognitions Questionnaire-65 Positive Beliefs scale Revised (MCQ-PBR; Meiser-Stedman et al., 2007).
7. The Cognitive Self Consciousness Scale-Expanded (CSC-E; Janeck et al., 2003).

The MCQ-A is a 30 item measure, based on the MCQ-30 with the wording simplified slightly with the aim of making it more understandable to adolescents. Like the MCQ-30 each item is scored on a scale of 1 (do not agree) to 4 (agree very much). Therefore, the possible range of scores for the total scale is 30–120, and for each subscale 6–30. It was used in 12 articles in this review, consisting of 11 separate population samples. Age range across studies was 7–18, although nine out of these 11 samples used adolescents of 12–18 years, the age group the questionnaire was originally devised for. English, French, Dutch, and Farsi versions of the MCQ-A were used in the studies.

The MCQ-C is a 24 item measure, based on the MCQ-A but with the wording further simplified with the goal of making it understandable to children as young as 7. An important difference between the MCQ-C and other versions of the MCQ is that the developers omitted the six items making up the cognitive confidence subscale. They justified omitting it based on a study that suggests that this scale in adults may be made up of different factors (Hermans et al., 2008) and they argued that it should be omitted until this was clarified. The removal of this subscale means the possible range of scores for the total scale of the MCQ-C is 24–90. It was used in 19 articles in the review, made up of 17 different samples, with an age range across the studies of 7–17. English, Turkish, Italian and Serbian versions of the MCQ-C were used.

The MCQ-CR is a 30 item measure. It was developed after Smith and Hudson (2013) tested the understandability of the MCQ-C in fourteen 7–8 year olds and found that a significant proportion of children did not understand six items. The MCQ-CR consists of 12 items from the MCQ-C without adaptation, as well as 12 more items taken from the MCQ-C and simplified further to be understandable to 7 and 8 year olds. The MCQ-CR reverted to the five-factor model of the MCQ and also included the six items of the cognitive confidence subscale, modified to make them understandable to children aged 7–8. The MCQ-CR adds an option for each item of indicating that the participant does not understand the item. The MCQ-CR was used by one study in the review (age range 7–12) and an English version was used.

The MCQ-C30 was based on the MCQ-A but with the wording simplified further to be understandable to children. Unlike the MCQ-C it retained the five-factor structure of the MCQ. It was used by eight studies in the review, consisting of five separate samples, age ranged from 7 to 17. The MCQ-C30 was originally developed in German, studies in the review used Danish or English versions of the questionnaire.

The MCQ-30 is the version developed in adults and is described earlier. It was used in two studies in the review without adapting it for younger participants, these studies had separate samples, ages in the two studies together ranged from 12 to 18. Both studies used English versions of the questionnaire.

The MCQ-PBR is a 19 item measure that consists of the positive beliefs about worry scale from the MCQ-65 with 10 items adapted to make them understandable to children. It was used by two studies with overlapping samples, age range 10–16. Both studies used English versions.

The CSC-E consist of 14 items and is an expanded version of the cognitive-consciousness scale of the MCQ-65. It was developed using an adult population (Janeck et al., 2003) but the English version of the CSC-E was used without adaptation by one study in the review with adolescents, ages 15–17.

### Symptoms Measured

Results extracted for tests of concurrent validity examined relationships between the MCQ measures and worry, anxiety, obsessive-compulsive symptoms, depression, post-traumatic symptoms, general emotional difficulties, psychotic symptoms and dissociation. Symptom measures for individual studies are given in Table 1.

### Assessment of Study Quality

Scores on the quality assessment scale (maximum possible 8) ranged from 2 to 7 with a mean of 5.13. All studies had clear research questions and appropriate design. Most studies used validated symptom measures and used appropriate analyses which were described appropriately. Studies varied as to the amount of possible bias in their sampling method with the strongest studies attempting to make their samples representative, by for example using schools in locations evenly spread across a country. Studies were marked down on sampling method if samples were clearly not representative or were at risk of not being representative e.g., using individual schools without discussing how representative these schools were. Few studies carried out power calculations. The adequacy of sample size was assessed by power calculations we made based on parallel adult studies, and studies varied as to whether they had adequate power according to these criteria. Missing data was only reported and addressed in a minority of studies.

### Metacognitions Questionnaire-Adolescent Version (MCQ-A)

#### Validity Evidence Based on Content

##### Comprehensiveness

The MCQ-A includes all items of the MCQ-30 with wording slightly simplified.

##### Understandability

Cartwright-Hatton et al. (2004) report that the MCQ-A has a Flesch-Kincaid Reading Grade Level of 3.6. This means the questionnaire should be understandable to most children aged 9 and up. Beyond this no other data is available regarding its understandability to younger populations.

#### Validity Evidence Based on Factor Structure

Three studies examined the factor structure of the MCQ-A. In their validation study, Cartwright-Hatton et al. (2004) carried out an exploratory factor analysis on a non-clinical sample (*n* = 158). They reported that a five-factor solution was chosen based on the Scree test and including only factors with Eigen values above one. The factors and item loadings corresponded closely with the adult MCQ-30, although goodness of fit indices were not reported. The Confirmatory Factor Analysis (CFA) of the MCQ-A carried out by Ellis and Hudson (2011) using a mixed clinical and non-clinical sample (total *n* = 114) found an adequate or good fit on four out of five fit indices: GFI = 0.96, AGFI = 0.95, NFI = 0.94, RFI = 0.94, PNFI = 0.86. Wolters et al. (2012) using a non-clinical sample (*n* = 317) found the five-factor structure had an adequate or good fit on all fit indices GFI = 0.95, AGFI = 0.94, NFI = 0.91, RFI = 0.90. Additionally, Wolters et al. found that a second-order model consisting of a higher-order factor (total score) and five lower-order factors (subscales) had an acceptable or good fit on most criteria. GFI = 0.94, AGFI = 0.93, NFI = 0.90, with the RFI of 0.89 just outside the criteria for acceptable fit. No studies examined measurement invariance across gender or age.

#### Internal Consistency

Internal consistency was examined in seven studies without overlapping participants (Cartwright-Hatton et al., 2004; Matthews et al., 2007; Ellis and Hudson, 2011; Wilson et al., 2011; Farrell et al., 2012; Wolters et al., 2012; Mazloom et al., 2016). Five of these studies examined the Cronbach alphas of the total score and subscales and a further two only examined total scores. Cronbach alphas were adequate to excellent for the total score and all subscales apart from NFC (range 0.70– 0.92). Results for the NFC were mixed, in three samples they were below the 0.7 threshold of adequacy (range 0.57–0.66) but Cronbach alphas were adequate in three other samples (range 0.70–0.77).

#### Test-Retest Reliability

Test-retest analysis was examined for the total score and subscales in three samples, in two papers (Cartwright-Hatton et al., 2004: 2 weeks test-retest; Wolters et al., 2012-Non-clinical sample, 7–21 weeks test-retest; OCD sample, 6–12 weeks test-retest). Results of intraclass correlations were mostly good to excellent (range 0.72–0.95) apart from poor reliability for the NB subscale (0.24) and the total score (0.34) in the former study and NFC (0.35) in the non-clinical sub-sample of the latter study.

#### Ranges

Three studies reported ranges for the MCQ-A (Cartwright-Hatton et al., 2004 [total score range only]; Matthews et al., 2007 and Wolters et al., 2012 [for clinicals and non-clinical participants separately]). Across-study ranges for the total score in non-clinical participants were 30–116. For subscales (across two studies) non-clinical ranges were PB 6–24, NB 6–24, CC 6–22, NFC 6–20, CSC 6–24. In the one study (Wolters et al., 2012) that reported ranges for clinical participants, for the total score, the range was 36–104, for subscales: PB 6–24, NB 6–22, CC 6–22, NFC 6–22, and CSC 7–24. Results suggested the measure picked up a broad range of MCQ scores.

#### Validity Evidence Based on Relations With Associated Measures

Nine studies with non-overlapping samples examined correlations between the MCQ-A and a range of psychological symptom measures. Results are shown in Table 2.

**Table 2:** Across-study correlations between MCQ-A and symptom measures.

**Measures**	**Obsessive-compulsive**	**Anxiety**	**Depression**	**Worry**	**Post-traumatic**	**Psychotic**
**MCQ-A**						
Total score	0.56^*^ to 0.69^*^	0.37^*^ to 0.62^*^	0.48^*^ to 0.53^*^	–	0.49^*^	0.54^*^
PB	0.36^*^ to 0.42^*^	0.19 to 0.47^*^	0.24^*^ to 0.46^*^	0.35^*^	–	0.31^*^
NB	0.52^*^ to 0.66^*^	0.32^*^ to 0.67^*^	0.38^*^ to 0.54^*^	0.74^*^	–	0.57^*^
CC	0.30^*^ to 0.36^*^	0.05 to 0.46^*^	0.38^*^ to 0.49^*^	0.16	–	0.38^*^
NFC	0.46^*^ to 0.58^*^	0.08 to 0.47^*^	0.27 to 0.44^*^	0.35^*^	–	0.39^*^
CSC	0.18 to 0.45^*^	0.29 to 0.41^*^	0.22 to 0.28^*^	0.42^*^	–	0.10

* *p < 0.05*.

*Number of samples with correlations between symptoms and MCQ-A Total Score (TS) and subscales—Obsessive-Compulsive symptoms: TS, five samples (Cartwright-Hatton et al., 2004; Matthews et al., 2007; Crye et al., 2010; Farrell et al., 2012; Wilson and Hall, 2012) subscales, three samples (Cartwright-Hatton et al., 2004; Matthews et al., 2007; Wilson and Hall, 2012); Anxiety: TS, three samples (Cartwright-Hatton et al., 2004; Wolters et al., 2012 - non-clinical group; Wolters et al., 2012 - OCD group) subscales, four samples (Cartwright-Hatton et al., 2004; Wolters et al., 2012 - non-clinical group; Wolters et al., 2012 - OCD group; Wilson and Hall, 2012); Depression: TS and subscales, three samples (Cartwright-Hatton et al., 2004; Wolters et al., 2012 - non-clinical group; Wolters et al., 2012 - OCD group); Worry: one sample (Wilson et al., 2011); Post-traumatic symptoms: one sample (Mazloom et al., 2016); Psychotic symptoms: one sample (Debbané et al., 2009 -controlling for age and IQ)*.

As shown the Total Score and the NB subscale significantly related to a range of symptoms in all analyses, with effect sizes ranging from medium to high. The other subscales related significantly to symptoms in most but not all analyses. Effect sizes for PB, CC, and CSC ranged from low to medium and for NFC from low to high.

#### Validity Evidence Based on Relations With a Criterion

Three studies (Cartwright-Hatton et al., 2004; Ellis and Hudson, 2011; Wolters et al., 2012) examined differences between the MCQ-A total score and subscales in clinical and non-clinical groups. The clinical groups consisted of people with “an emotional disorder” not specified (Cartwright-Hatton et al., 2004), anxiety disorders or anxiety disorders with comorbid depression (Ellis and Hudson, 2011) and Obsessive-Compulsive Disorder (Wolters et al., 2012). In all three studies the total score (range *d* = 1.06 to 1.49) and the NB (range *d* = 1.54 to 2.41) and NFC (range *d* = 0.61 to 1.00) subscales were significantly higher in clinical groups than control groups-effect sizes high for total score and NB, medium to high for NFC. PB was higher in two out of three studies (range *d* = 0.19 to 0.67) while CSC (range *d* = 0.31 to 0.78) and CC (range *d* = 0.20 to 1.03) were both higher in one out of three studies.

#### Responsiveness

One study (Sanger and Dorjee, 2016) reported on changes in MCQ-A scores following an intervention, which consisted of a course of mindfulness training in non-clinical adolescents. The intervention was successful in leading to significantly increased response inhibition as shown by increased N2 negativity response to an attention task measured by an EEG. However, it did not lead to predicted changes on P300 mean amplitude (measures of attention efficiency). Significant pre to post differences were found on the total score of the MCQ-A (*d* = 0.64, medium effect size) as well as on NFC (*d* = 1.15, large effect size) compared to a control group, but not on the other subscales.

#### Age

Four articles tested whether there were within-study relationships between age of participants and the MCQ-A total score and subscales: Matthews et al. (2007) in a non-clinical sample, age range 13–16, found that the MCQ-A total score, as well as the NB, NFC, and CSC subscales significantly negatively correlated with age, although correlations were low (range −16 to −19). Wilson et al. (2011) using a non-clinical sample, age range 11–16, and Ellis and Hudson (2011) using a mixed non-clinical and clinical sample, age range 12–17, found no significant correlations between age and the total score or subscales. Wolters et al. (2012), age range 12–18, also found no relationship between the total score or subscales and age in their clinical sample. In their non-clinical sample, there was a small positive relationship between the MCQ-A total scale and age, *r* = 0.12.

#### Differences Between Sexes

Three studies examined differences between sexes in MCQ-A scores. Matthews et al. (2007) and Wilson et al. (2011) using the MCQ-A total score and subscales, Crye et al. (2010) using the MCQ-A total score only. No significant differences were found on any scores.

### Metacognitions Questionnaire-Child Version (MCQ-C)

#### Validity Evidence Based on Content

##### Comprehensiveness

The MCQ-C does not include the six items designed to assess cognitive confidence in the MCQ-30. Bacow et al. (2009) justified omitting it based on the fact that this scale in adults may be made up of different factors (Hermans et al., 2008) and they argued that it should be omitted until this was clarified. Thus, one factor assessed in the MCQ-30 is not assessed in the MCQ-C.

##### Understandability

Bacow et al. (2009) report that the MCQ-C has a Flesch-Kincaid Reading Grade Level of two-meaning it should generally be understandable to children ages 7–8. However, Smith and Hudson (2013) tested the understandability of the questionnaire in a sample of fourteen 7–8 year olds and found that a significant proportion of these children did not understand six items on the MCQ-C. Additionally, White and Hudson (2016) reported that six further items were assessed as being above Grade 2 level according to Fry's (1977) criteria.

#### Validity Evidence Based on Factor Structure

Three studies (Bacow et al., 2009; Irak, 2012; Stevanovic et al., 2016) examined the factor structure of the MCQ-C. Both Bacow et al. in a combined clinical and non-clinical sample (*n* = 98) and Irak using a large non-clinical sample (*n* = 470), carried out a confirmatory factor analysis of the four-factor structure of the MCQ-C. Results of fit indices in Bacow et al.'s were mixed with an RMSEA of 0.077 suggesting an adequate fit but a CFI of 0.85 suggesting a poor fit. Irak's fit indices were good for RMSEA = 0.05 and RMR = 0.08, adequate for GFI = 0.90 and just marginally below adequate for CFI = 0.89 and AGFI = 0.88. Stenanovic et al. split their sample (*n* = 467) into two, with both of these samples having mixed clinical and non-clinical participants. They first carried out an exploratory factor analysis of the MCQ-C on one part of the sample (*n* = 233). This resulted in a three rather than four factor structure, made up of 16 items in total described as: (1) Cognitive monitoring, (2) Specific positive worry beliefs, and (3) General positive worry beliefs. In a subsequent CFA using the second part of their sample (*n* = 234) testing this three-factor structure, an adequate fit was obtained when three items of one of the scales were removed. No studies reported examining measurement invariance based on gender or age.

#### Internal Consistency

Eleven articles (Bacow et al., 2009; Irak, 2012; Smith and Hudson, 2013; Benedetto et al., 2014; Holmes et al., 2014; Carr and Szabó, 2015; Donovan et al., 2017; Francis et al., 2017, 2018; Hearn et al., 2017a), representing 10 separate samples reported Cronbach alphas for the MCQ-C (two studies Francis et al., 2017 and Francis et al., 2018 reported Cronbach alphas for different parts of the scale in the same sample). Internal reliability of the MCQ-C total score was adequate to good in the three studies that reported it (range 0.73 to 0.87). Scores on subscales varied depending on the study PB (nine studies) range 0.46 to 0.86, NB (eight studies) range 0.60 to 0.78, NFC (three studies) 0.25 to 0.64, CSC (three studies) 0.61i to 0.75.

#### Test-Retest Reliability

Test-retest reliability, over a 3 week period, reported only by Irak was good to excellent for all subscales and the total score (range 0.76 to 0.82).

#### Ranges

Two studies with the same sample gave ranges for the MCQ-C (Francis et al., 2017, total score; Francis et al., 2018, PB and NB subscales). Ranges were broad: Total score ranged from 26 to 79, PB 6 to 22 and NB 6 to 24.

#### Validity Evidence Based on Relations With Associated Measures

Eleven studies with non-overlapping samples examined correlations between the MCQ-C and a range of psychological symptom measures. Results are shown in Table 3.

**Table 3:** Across-study correlations between MCQ-C and symptom measures.

**Measures**	**Obsessive-compulsive**	**Anxiety**	**Depression**	**Worry**	**Post-traumatic**	**Dissociation**	**Emotional difficulties**
**MCQ-C**							
Total score	0.40^*^, 0.59^*^	0.40^*^ to 0.48^*^	0.33^*^, 0.25^*^	0.48^*^	0.41^*^	0.47^*^	0.45^*^
PB	0.19^*^, 0.39^*^	0.02 to 0.30^*^	0.04	0.16 to 0.39^*^	–	–	0.28^*^
NB	0.33^*^, 0.47^*^	0.39^*^ to 0.65^*^	0.36^*^	0.50^*^ to 0.72^*^	–	–	0.48^*^
NFC	0.24^*^, 0.45^*^	0.19 to 0.42^*^	0.13	0.33^*^	–	–	0.18
CSC	0.27^*^, 0.45^*^	0.11 to 0.27^*^	0.17^*^	0.30^*^	–	–	0.21

* *p <0.05*.

*Number of samples with correlations between symptoms and MCQ-C Total Score (TS) and subscales—Obsessive-Compulsive symptoms: TS and subscales, two samples (Irak, 2012; Boysan et al., 2016); Anxiety: TS, three samples (Irak, 2012; Kadak et al., 2013; Smith and Hudson, 2013) PB and NB four samples (Irak, 2012; Smith and Hudson, 2013; Benedetto et al., 2014; Hearn et al., 2017a) NFC and CSC, three samples (Irak, 2012; Smith and Hudson, 2013; Benedetto et al., 2014); Depression: TS, two samples (Bacow et al., 2009; Kadak et al., 2013) subscales, one sample (Bacow et al., 2009); Worry: TS, one sample (Bacow et al., 2009) PB, six samples (Hearn et al., 2017a; Bacow et al., 2009; Kertz and Woodruff-Borden, 2013; Carr and Szabó, 2015; Donovan et al., 2017; Francis et al., 2018, controlling for recruitment site) NB, five samples (Bacow et al., 2009; Kertz and Woodruff-Borden, 2013; Donovan et al., 2017; Hearn et al., 2017a; Francis et al., 2018, controlling for recruitment site) NFC and CSC, one sample (Bacow et al., 2009); Post-traumatic symptoms: one sample (Kadak et al., 2013); Dissociation: one sample (Kadak et al., 2013); Emotional Difficulties: one sample (Smith and Hudson, 2013)*.

As shown the Total Score and the NB subscale significantly related to a range of symptoms in all analyses. Effect sizes for the total score ranged from low-medium to high and for NB from medium to high. The other subscales related significantly to symptoms in most but not all analyses with effect sizes ranging from low to medium.

#### Validity Evidence Based on Relations With a Criterion

Four studies examined differences between clinical and non-clinical populations: [Bacow et al., 2009; Smith and Hudson, 2013 using clinical groups with anxiety disorders; Donovan et al. (2016) using a group with Generalized Anxiety Disorder (GAD), and Hearn et al. (2017b) using a group with Social Anxiety Disorder, the GAD comparison in Hearn et al. was not included as the GAD sample overlapped with Donovan et al. (2016)]. Smith and Hudson found significantly higher scores in the clinical than the non-clinical group for the total score (*d* = 0.69), PB (*d* = 0.45) (medium effect sizes), and NB (*d* = 0.87; large effect size). However, NFC and CC did not distinguish the groups. Donovan et al. and Hearn et al. examined only NB and PB, both found that NB (*d*s of 1.72 and 1.15; both large effects) but not PB (*d*s of 0.52 and 0.25) was significantly higher in the clinical group than a non-clinical control. Bacow et al. (2009), with worry content controlled, found no significant differences between a clinical and non-clinical group on the total score or subscales beyond significantly higher scores on CSC in the non-clinical group. Of note in this study was that 60% of the non-clinical group had sub-clinical symptoms.

#### Responsiveness

Holmes et al. (2014) in a trial treating GAD using Cognitive Behavior Therapy (CBT), in children aged 7–12, examined changes on only the NB and PB subscales of the MCQ-C. Examination of primary outcome measures showed the treatment was successful in reducing diagnostic GAD status and severity of disorder post-treatment compared to a control group, as well as leading a larger increase in overall functioning. They found a significant decrease in NB but not PB from pre-treatment to both post-treatment and 3 month follow up, effect size not reported. However, the decrease in NB was not significantly different from the decrease seen in a wait list control, assessed only at post-treatment.

Hearn et al. (2018) in a trial of CBT with patients with Social Anxiety Disorder (age of participants 8–17) also examined changes on only the NB and PB of the MCQ-C. They examined scores on measures at 12 week assessment when some but not all participants had completed treatment and at 6 month follow-up. At 12 week assessment there were significant reductions in diagnostic severity and social anxiety symptoms in the treatment group compared to the wait-list control. However, there were no significant difference on diagnostic status. They found no significant reductions at 12 week assessment on NB and PB. However, there were significant reductions from pre-treatment to 6 month follow-up in both NB and PB.

#### Age

Three articles examined relationships between scores on the MCQ-C and age. Bacow et al. (2009), age range 7–17, examined age differences in their clinical group only, due to the small sample size of their non-clinical group. Of the four subscales and total score of the MCQ-C the only significant relationship was a positive relationship between CSC and age (only the unstandardized regression coefficient is reported (0.46). Irak (2012) split his sample into children (age 8–12) and adolescents (13–17). There was a significant difference between the groups on PB scores only, with the older group scoring higher. Carr and Szabó (2015) in a non-clinical sample, age range 7–12, examined only PB and found no relationship between this subscale and age.

#### Differences Between Sexes

Three studies examined differences in MCQ-C scores (Benedetto et al. using the MCQ-C subscales, Francis et al., 2018 using just the PB and NB subscales of the MCQ-C, and Irak, 2012, using the MCQ-C total score and subscales). Benedetto et al. and Francis et al. found no differences between scores of males and females. Irak found that females scored significantly higher than males on negative beliefs about worry, and the total score only.

#### Age X Gender Interaction

Two studies examined the interaction between age and gender; both studies used the MCQ-C total score and subscales. bib4 (2009; age range 7–17) found that for younger participants (1 SD below mean age) there were no gender differences on MCQ-C subscales or total score. However, in adolescents (1 SD above mean age) girls scored higher than boys on the MCQ-C total score only. bib44 (2012; age range 8–17) found no interaction effect between age and gender on the total score or subscales.

### Metacognitions Questionnaire-Child 30 (MCQ-C30)

#### Validity Evidence Based on Content

##### Comprehensiveness

The MCQ-C30 includes all items of the MCQ-30 with wording simplified to be understandable to children.

##### Understandability

No data on reading level or understandability was presented for this measure.

#### Validity Evidence Based on Factor Structure

One study (Esbjørn et al., 2013) examined the factor structure of the MCQ-C30. This study carried out a CFA examining the fit of a two-level model with the higher-order factor consisting of the total score and the five subscales making up lower-order factors. They also included gender as a predictor of the total score. In their full non-clinical sample (*n* = 974) fit indices for the model were acceptable or good: CFI = 0.94, TLI = 0.93, RMSEA = 0.039. They subsequently carried out two CFAs on their sample split by age. This is a test of measurement invariance across age. Results for 13–17 year olds (*n* = 420) indicated an adequate fit to the two-level model: CFI = 0.90, TLI = 0.90, RMSEA = 0.06, while results for the 9–12 year olds (*n* = 554) were acceptable on one measure: RMSEA = 0.06, but marginally short on two others: CFI = 0.87, TLI = 0.86. Tests comparing the model fit of the two age groups showed no significant differences between them.

#### Internal Consistency

Five studies with non-overlapping samples (Esbjørn et al., 2013, 2015; Campbell et al., 2018 only results for clinical sample included for this study as non-clinical sample overlapped with another study Esbjørn et al., 2016, 2018) reported Cronbach alphas. Cronbach alphas for the total score ranged from just below the adequate cut-off to excellent: range 0.69–0.91. Subscale scores were somewhat mixed: PB 0.64–0.87, NB 0.65–0.78, CC 0.66–0.82, CSC 0.62–0.75^1^. NFC generally showed the weakest Cronbach alphas, with scores ranging from 0.59 to 0.68.

#### Test-Retest Reliability

No studies using this measure reported test-retest results.

#### Ranges

Ranges for the MCQ-C30 were not presented in any study.

#### Validity Evidence Based on Relations With Associated Measures

Three studies with non-overlapping samples examined correlations between the MCQ-C30 and psychological symptom measures. One of these studies (Campbell et al., 2018) had a very small sample (*n* = 23) which meant even some medium effect sizes were not significant in this study. Studies using both the Total score and subscales of the MCQ-C30 examined relationships with anxiety, depression and worry.

Results are shown in Table 4.

**Table 4:** Across-study correlations between MCQ-C30 and symptom measures.

**Measures**	**Anxiety**	**Depression**	**Worry**
**MCQ-C30**			
Total score	0.81^*^^a^, 0.51^*^^b^, 0.66^*^^c^	0.47^*^^a^	0.37^*^^b^, 0.37^*^^c^
PB	0.36^a^, 0.17^*^^b^	0.46^*^^a^	0.25^*^^b^
NB	0.68^*^^a^, 0.55^*^^b^	0.39^a^	0.31^*^^b^
CC	0.35^a^, 0.30^*^^b^	−0.05^a^	0.27^*^^b^
NFC	0.64^*^^a^, 0.43^*^^b^	0.33^a^	0.33^*^^b^
CSC	0.20^a^, 0.36^*^^b^	0.21^a^	0.25^*^^b^

* *p <0.05*.

a *(Campbell et al., 2018)*.

b *(Esbjørn et al., 2013)*.

c *(Esbjørn et al., 2016)*.

As shown the Total Score significantly related to symptoms in all analyses with effect sized ranging from medium to high. NB and NFC significantly related to anxiety and worry but not depression with all effect sizes ranging from medium to high. PB significantly related to symptoms in three out of four analyses and CC and CSC in two out of four with effect sizes for these subscales ranging from low to medium.

#### Validity Evidence Based on Relations With a Criterion

Only one study examined differences between clinical and non-clinical groups with Esbjørn et al. (2015) finding that a group with Generalized Anxiety Disorder scored significantly higher on all subscales apart from CSC: PB (*d* = 0.70), NB (*d* = 1.58), CC (*d* = 0.69), and NFC (*d* = 1.07; range of effect sizes for significantly different scores medium to large) and that an Anxiety Disorder group scored significantly higher on NB (*d* = 1.15) and NFC (*d* = 0.87) than a non-clinical group (both large effect sizes).

#### Responsiveness

Two studies examined changes in MCQ-C30 scores following treatment. Esbjørn et al. (2018) in a trial of MCT for GAD in participants aged 7–13 found 86.4% were free of GAD and 72.7% were free of all anxiety disorders post-treatment, at 6 month follow-up figures were 75 and 65.9%, respectively. The total score of the MCQ-C30 and all subscales apart from CC, were significantly reduced from pre to post-treatment, and all reductions remained significant at 6 months follow-up apart from PB. The effect-size, reported for the total score only, was large both from pre to post treatment, *d* = 0.84, and from pre-treatment to follow up, *d* = 1.08.

Normann et al. (2016) in a trial of CBT for patients aged 7–12, with several anxiety disorders, examined changes on the total score of the MCQ-C30. The treatment successfully reduced anxiety symptoms from pre-treatment to post-treatment (medium effect) and pre-treatment to follow-up (large effect). The MCQ-C30 Total Score changed significantly from pre-treatment to post-treatment *d* = 0.55 (a medium effect size), and from pre-treatment to follow-up *d* = 0.87 (large effect size). There was a significant decrease from post-treatment to follow-up.

#### Age

One study (Lønfeldt et al., 2017b; age range 9–17) using the MCQ-C30 examined relationships between the total score and subscales and age. They found small but significant negative relationships between the MCQ-Total score (−0.08) as well as NB (−0.08) and NFC (−0.10) and age, for other subscales the relationship was not significant.

#### Differences Between Sexes

Two studies examined differences on the MCQ-C30. Esbjørn et al. (2013) found a small but significant correlation between gender and the total score (subscales not examined), with girls scoring higher, but this difference was made non-significant when anxiety was controlled for Lønfeldt et al. (2017a) found that NB but not other scales or the total score were significantly higher in girls than boys.

### Metacognitions Questionnaire-Child Revised (MCQ-CR)

This questionnaire has only been examined in its validation study (White and Hudson, 2016) results are outlined below.

#### Validity Evidence Based on Content

##### Comprehensiveness

The MCQ-CR includes all items of the MCQ-30 with wording simplified to be understandable to children as young as 7–8.

##### Understandability

The MCQ-CR includes the possibility of indicating “I don't understand” for each item Examination of responses suggested 75% of 7 year olds and 83% of 8 year olds understood all items on the MCQ-CR. However, there was a negative correlation (*r* = −0.23) between number of items filled in as “I don't understand” and age, indicating that understanding increased with age. A *t*-test comparing 7–8 year olds with 9–12 year olds found significantly greater lack of understanding in the younger group. For other analyses in the White and Hudson (2016) study items scored as “I don't understand” were treated as missing data. If only one item of a subscale was missing, data was replaced by the mean of that subscale, if more items were missing they were deleted pairwise or listwise depending on whether used in bivariate or multivariate analyses.

#### Validity Evidence Based on Factor Structure

In a CFA testing the five-factor structure, the RMSEA result 0.06 was acceptable while IFI (0.89) and TLI (0.87) were just under the acceptable criteria.

#### Internal Consistency

Cronbach scores for the subscales and total score were adequate to excellent-range 0.76–0.90.

#### Test-Retest Reliability

White and Hudson did not explore test-retest and this is currently unknown.

#### Ranges

Ranges for the MCQ-CR were broad: Total score 30–104, PB 6–22, NB 6–24, CC 6–23, NFC 6–24, CSC 6–23.

#### Validity Evidence Based on Relations With Associated Measures

White and Hudson examined correlations between the MCQ-CR and both anxiety and worry. There were significant positive relationships between the total score and all subscales of the MCQ-CR and anxiety, with large effect sizes for Total score *r* = 0.56 and NB *r* = 0.56, medium effect sizes for CC *r* = 0.31, NFC *r* = 0.47, and CSC *r* = 0.46, and a small effect size for PB, *r* = 0.20. The Total score (*r* = 0.55) and NB (*r* = 0.65) also were significantly related to worry with large effect sizes, and NFC (*r* = 0.46) and CSC (*r* = 0.48) significantly related to worry with medium effect sizes. PB (*r* = 0.08) and CC (*r* = 0.13) were not significantly correlated with worry.

Criterion-based validity evidence, and responsiveness were not tested.

#### Age

The White and Hudson (2016) study had an age range of 7–12. They found a significant negative correlation between age and CSC (*r* = −0.36) and NFC (*r* = −0.15), relationships with other subscales and the total score were not significant.

#### Differences Between Sexes

Differences on the total score and subscales were examined and all were non-significant.

### Other MCQ Measures

There was less comprehensive psychometric data available for other MCQ measures used. The two studies that used the MCQ-30 (Gallagher and Cartwright-Hatton, 2008; Welsh et al., 2014) did not report level of understandability, factor-analysis data, internal consistency, test-retest data, range, or analysis of age and gender relationships. Concurrent-based evidence of validity of the MCQ-30 total score came from Gallagher and Cartwright Hatton's finding that it significantly correlated with anxiety, only unstandardized betas were reported. Criterion based evidence for validity of the total score and some subscales came from Welsh et al.'s finding that a group, aged 12–17, at high risk of psychosis, scored significantly higher on the total score (*d* = 1.16), NB (*d* = 1.49), CC (*d* = 0.93) and NFC (*d* = 0.92) than controls, all effect sizes were large.

The positive beliefs about worry subscale of the MCQ-65 (MCQ-PBR) adapted for children by Meiser-Stedman et al. (2007) had excellent internal consistency (0.90). Concurrent based validity was supported by the fact that it significantly correlated with a measure of trauma symptoms cross-sectionally (*r* = 0.34). It also significantly correlated prospectively with trauma symptoms 6 months after the trauma (*r* = 0.38) but this relationship became non-significant when time 1 trauma symptoms were controlled for (Meiser-Stedman et al., 2009). Criterion based evidence of validity came from the fact that scores on the MCQ-PBR were significantly higher in a group with Acute Stress Disorder than a control group (*d* = 0.66; a medium effect size) (Meiser-Stedman et al., 2007).

The CSC-E used by Jacobi et al. (2006) had adequate internal consistency (0.77). Concurrent-based evidence for validity was shown by significant relationships between the CSC-E and measures of obsessive-compulsive symptoms, anxiety and depression, individual *r*s not given. Criterion-based validity evidence was not assessed. The MCQ-PBR and CSC-E are purportedly unidimensional but this was not tested in these studies nor was level of understandability, test-retest data, range, or age and gender differences discussed.

## Discussion

### Overview

Forty-five studies that used MCQ measures or derivatives in children/adolescents were identified in the review reflecting the growth in this research area. Studies used one of seven versions of MCQ measures or derivatives. Of these, one was adapted from the MCQ for use in adolescents-the Metacognitions Questionnaire-Adolescent version (MCQ-A); four for use with children: Metacognitions Questionnaire-Child version (MCQ-C), Metacognitions Questionnaire Children-30 (MCQ-C30), Metacognitions Questionnaire-Child version Revised (MCQ-CR) and the Metacognitions Questionnaire-65 Positive Beliefs scale Revised (MCQ-PBR); and two measures developed for adults were used without adaptation: Metacognitions Questionnaire-30 (MCQ-30), and the Cognitive Self Consciousness-Expanded scale (CSC-E).

The MCQ-A (12 studies) and MCQ-C (19 studies) were the most commonly used and the largest amount of psychometric data is available for these measures. The MCQ-C30 was used in eight studies but these consisted of only five separate samples. Other MCQ measures were each used in two or less studies.

Most studies using the MCQ-A only recruited adolescent participants (aged 12 and older)-the age group that the questionnaire was designed-for, so psychometric data for the MCQ-A largely represents the measure's properties as used with adolescents. Of the four measures designed for use with children, studies examining the MCQ-C, MCQ-C30, and MCQ-PBR used participants with a range of ages spanning children and adolescents (range across measures 7–18), while the one study that examined the MCQ-CR used children aged 7–12. Studies that used adult measures-the MCQ-30 and CSC-E-used adolescent samples and so results reflect their use with this population.

### Factor Structure

The strongest evidence supporting factor structure and latent constructs they represent exists for the MCQ-A as its five-factor structure was supported in the three studies that examined it (Cartwright-Hatton et al., 2004; Ellis and Hudson, 2011; Wolters et al., 2012). However, some caution must be applied when interpreting these results as only one of these studies (Ellis and Hudson, 2011) included clinical populations in their sample and only one of these studies (Wolters et al., 2012) had a sample >300. While all three studies examined a single-order model consisting of the five subscales of the adult versions of the MCQ, only Wolters et al. also examined a second-order model with total score as the higher-order factor and the five-subscales as lower order factors. Results suggested an adequate fit reflecting a recent study in adults which found that the data supported the MCQ-30 as having a second-order or bifactorial model in a large adult population (Fergus and Bardeen, 2017). Initial factor-analysis results of the MCQ-A are promising and were in line with our hypothesis that the factor structure of MCQ in younger populations would be similar to the one found in adults. However, further studies of both single and second-order models are warranted, particularly using large clinical populations. Studies examining measurement invariance of the MCQ-A factors across gender and age are needed as this has not yet been assessed.

Evidence for the four-factor structure of the MCQ-C, examined in three studies, was mixed. The removal of the cognitive confidence subscale, one of the factors present in earlier versions of the MCQ, from the MCQ-C means that participants were not exposed to the same items as those who completed the full 30 item version in other studies. It is possible that this led to somewhat different responses in the retained items and it is also possible that this could affect item clustering and latent variables emerging from factor-analyses. An additional problem with removing one subscale from the measure is that one important type of metacognition identified in the S-REF model is not assessed. It also prevents comparison of results on this subscale with results from other versions of the MCQ. A strength of other MCQ full-scale measures in contrast is that their structure and items match the MCQ-30, allowing comparison of analyses using all subscales and the total score from children to adolescents to the adult population. The reasoning given by Bacow et al. (2009) for removing the cognitive confidence factor was that results from a study suggested that cognitive confidence may comprise several different elements–confidence in memory, reality monitoring and attention (Hermans et al., 2008) and that they wished to remove this factor until further research clarifies this. However, in our view this does not justify dropping this subscale from the questionnaire.

The five-factor structure of the MCQ-C30, with the total score as a higher-order factor, was supported, particularly in 13–17 year olds, but was only tested in one study and this study used a non-clinical population. Further studies examining the MCQ-C30's factor structure and measurement invariance using clinical and non-clinical populations are needed.

The MCQ-CR was only used in one study (White and Hudson, 2016). The factor-analysis results examining a five-factor structure was only partially supportive of its latent structure. The MCQ-CR introduced the possibility of responding “I don't understand” to each item and the impact of this on factor-analysis and other results needs to be considered. An advantage of having the possibility of giving this response is that it can help in assessing which items are not well-understood. However, a significant disadvantage is that it introduces a new response to each item, that is not part of the original measure, which might bias interpretation and the desired response to the items. For example, rather than completing items based on the first overall impression, the person is asked to analyze their own understanding or doubts about the meaning of items in this context which may introduce deliberation and bias responses. Additionally, it raises the question as to how to treat items scored as “I don't understand.” In the White and Hudson study they were treated as missing data which, depending on the amount of missing items, was replaced by means or deleted. A problem with this is that certain items may have not been generally understood more than others and so the pattern of missing data may not have been random.

The factor structure of the other MCQ measures i.e., the MCQ-30, MCQ-PBR, and CSC-E were not examined in the studies included in the review and remain to be explored in children/adolescent populations.

### Internal Reliability

The internal validity of most subscales and the total score of the MCQ-A were supported by adequate to excellent Cronbach alphas across studies although evidence for the internal reliability of the Need for Control subscale was mixed and needs further exploration. Of note, in the validation study of the MCQ-30 in adults the NFC subscale had the poorest internal reliability (Wells and Cartwright-Hatton, 2004). The internal reliability of the MCQ-C total score was supported in the three studies that examined it. The internal reliability of individual subscales varied between studies, with PB and NFC in particular having weak internal consistency in certain studies but not others. There was a similar pattern with the MCQ-C30 with general support for the total score but variations on subscales, with NFC having the lowest range of Cronbach alphas. Internal validity of the MCQ-CR Total score and subscales, MCQ-PBR and CSC-E were supported but were only examined in one study each and further exploration is needed.

### Validity Evidence Based on Relations With Associated Measures

Concurrent-based evidence for validity was strong across MCQ measures used, with significant relationships demonstrated between the different measures and a range of psychological symptoms. As per our hypothesis, of the subscales, the strongest and most consistent results were for NB. NB correlated significantly with a range of symptoms in almost all analyses across MCQ measures and all correlations represented medium or large effect sizes using Cohen's criteria. Results for NB reflect findings using the MCQ-30 in adults where NB relates strongly to a range of symptoms (e.g., Wells and Cartwright-Hatton, 2004; Spada et al., 2008; Bailey and Wells, 2013). This is consistent with the central role of beliefs concerning the uncontrollability and danger of thoughts in prolonging and intensifying psychological difficulties in the S-REF model (Wells and Matthews, 1994). The total score also emerged as a consistent predictor of symptoms, in fact it significantly related to different symptoms in every analysis that used it across MCQ measures. The strong and consistent findings for the total score may indicate the importance of a general metacognitive factor across disorders, while some of the subscales apart from NB may have more variability as to their levels of importance depending on which type of psychological difficulty.

### Validity Evidence Based on Evidence of Relations With a Criterion

Criterion-based evidence of validity of the total score and NB and NFC subscales of the MCQ-A came from findings in three studies that these scores were consistently higher in clinical than non-clinical groups, all with large or medium effect sizes, results for other subscales were less consistent. NB and NFC also distinguished clinical and non-clinical groups in the one study that tested this using the MCQ-C30 and along with CC in a study using the MCQ-30 (Welsh et al., 2014). Results reflect our hypothesis that, of the subscales, NB and NFC would most consistently and strongly differentiate clinical and non-clinical groups. This parallels findings in adults, with a meta-analysis examining across-study differences on MCQ subscales between clinical and non-clinical groups finding that the negative beliefs and need for control subscales were highest in clinical groups when compared to non-clinical controls, with large effect sizes (Sun et al., 2017). In studies that used the MCQ-C, the NB subscale was significantly higher in clinical than non-clinical groups in three out of four studies. However, the NFC subscale did not emerge as significantly higher in the two studies that examined this although in one of these studies most of the comparison non-clinical group had sub-clinical symptoms which in a fairly small sample was likely to have obscured results. The number of studies comparing clinical and non-clinical children/adolescents across MCQ measures is relatively small and further comparisons are needed particularly as in the meta-analysis of a large number of adult studies, all MCQ subscales emerged as significantly higher in clinical compared to non-clinical groups.

### Responsiveness

Only five studies in the review examined changes in MCQ scores following an intervention or treatment. Studies examining MCQ scores following CBT or Mindfulness interventions using the MCQ-A, MCQ-C, and MCQ-C30 found decreases on at least some subscales and/or total score giving initial support for some responsiveness for these measures. These results were in line with our hypothesis that there should be some change in MCQ scores following any form of treatment that was successful in reducing symptoms. The only study in the review (Esbjørn et al., 2018) that carried out a trial of Metacognitive Therapy (MCT), used the MCQ-C30 as one of their outcome measures. This is a particular test of responsiveness as MCT for GAD, examined in this study, attempts to modify a number of the belief domains measured by the MCQ. The findings of large effects for decreases on the total score, and significant decreases in most subscales at post-treatment is a promising finding for the use of the MCQ-C30 to measure changes in metacognitions following treatment in young populations. Results are consistent with our hypothesis that changes in MCQ scores would be particularly apparent following MCT. The responsiveness of the MCQ-C30 was also supported by a CBT trial which examined changes in the total score of the MCQ-C30 and found medium effects at post-treatment and large effects at follow-up. Further studies of responsiveness of the different MCQ measures, particularly following MCT, are needed.

### Age

Studies that examined the relationships between age and the MCQ-A total score and subscales (age range across studies 11–18) found either no or small relationships. This is supportive of the idea that these metacognitions could be fully formed as early as 11 and remain stable across adolescence. However, studies did not break down the distribution of ages within their studies. Findings with other MCQ measures, that included younger participants, were somewhat mixed with individual subscales emerging in only some analyses as being related to age either positively or negatively, using the MCQ-C, MCQ-C30, and MCQ-CR. To fully test if there are any age differences in MCQ scores between children/adolescents of different ages, future studies should consider recruiting participants with an even distribution of age, or directly comparing scores of groups of younger and older children. The one study in the review that did the latter (Irak, 2012) found that 13–17 had higher scores on the positive belief subscale only compared to 8–12 year olds, further studies are needed to see if this result is replicated. Current findings, together with the fact that ranges of MCQ measures when given were broad, suggest that dysfunctional metacognitions could develop at an early age. This is consistent with findings that suggest there may be childhood factors that lead to vulnerability to the development of these metacognitions, such as early experiences of emotional abuse (e.g., Myers and Wells, 2015; Østefjells et al., 2017) and parenting style (Gallagher and Cartwright-Hatton, 2008; Spada et al., 2012; Lønfeldt et al., 2017b).

### Differences Between Sexes

Most studies that examined differences between males and females on scores of MCQ measures (MCQ-A, MCQ-C, MCQ-C30, MCQ-CR) did not find significant differences which suggest they may, as hypothesized, not be present or may be small. Of note in one of the minority of studies that found differences (Esbjørn et al., 2013; a significantly higher score for girls on the total score) was that controlling for anxiety removed the effect, suggesting it may have been caused by elevated anxiety symptoms in girls. As higher prevalence rates for having an anxiety disorder in females compared to males have been found in children (Anderson et al., 1987); adolescents (Lewinsohn et al., 1998), and adults (Kessler et al., 1994) it may be important for future studies to control for anxiety in analyses of sex differences on MCQ scores in all these groups.

### Test-Retest Reliability

Test-retest reliability was examined in few studies using any of the MCQ measures but results with the MCQ-A and MCQ-C mainly support the stability of the measures over time. More research is needed into this across measures.

### Understandability

The understandability of the measures also needs further investigation. No study, to our knowledge, has examined the understandability of the MCQ-A to adolescents or pre-adolescents or whether the MCQ-A is more understandable to adolescents than the MCQ-30 and there is a need for these issues to be investigated. The understandability of the MCQ-C30, MCQ-PBR, and CSC-E to children or adolescents has also not been examined, while the one study that examined the understandability of the MCQ-C found that six items were not understandable to most of the small sample of 7–8 year olds tested. Although the MCQ-CR was found to be understandable to most 7–8 year olds in the one study that used it, understanding increased with age.

## Conclusions

The choice of which version of the MCQ to use in future studies in younger populations may well be influenced by the age group of the population being examined. The MCQ-A has largely good psychometric parameters in adolescents, the population it was designed for, and few studies have used it with younger populations. We suggest future studies using adolescents should certainly consider using the MCQ-A. Studies whose participants include pre-adolescent children and who want to measure the full range of constructs measured by the MCQ-30 should consider using the MCQ-C30 which has initial, although currently relatively limited, psychometric data supporting it. Two studies suggest that the MCQ-C30 is responsive to changes in metacognition following treatment and so the MCQ-C30 may be a particularly appropriate choice for treatment trials that include children.

The youngest age of children included in studies in the review was seven and psychometrics for children younger than this are unknown. The fact that studies only recruited children aged seven and above reflects the traditional view that this is the age where children can report on metacognitive knowledge (Flavell, 1979). However, a recent study (Marulis et al., 2016) suggests that when measured appropriately some younger children-age 3-5 may be able to report on their metacognitions. Although not using an MCQ measure (Wilson and Hughes, 2011), found that some 6 year olds held both positive and negative metacognitions about worry. Future studies may consider examining children younger than seven on MCQ measures although content and means of administration may well have to be adapted further to accommodate this group.

Strengths of almost all studies reviewed include clearly stated aims/hypotheses, the use of standardized symptom or diagnostic measures and appropriate analyses. Studies varied as to the appropriateness of selection criteria and the adequacy of sample size. Only a minority of studies discussed and corrected for missing data. Although the quality of studies was generally good, the methodological limitations, in particular variable sample sizes, should be born in mind when interpreting psychometric results. A number of studies included younger children and as results from two studies suggest some younger children may have difficulty in understanding some MCQ items, caution must be applied in interpreting some psychometric results of these studies. Although a number of studies included clinical populations most used non-clinical populations thus psychometrics for non-clinical groups are more extensive. No studies carried out analyses of psychometrics based on Item Response Theory (IRT) which has a number of advantages over analyses based on Classic Test Theory. Future studies would be strengthened by carrying out psychometric analyses based on IRT.

Bearing in mind these limitations, this review suggests that several MCQ measures have promising psychometrics in younger populations. The metacognitions assessed by the MCQ appear to be present in children/adolescents and can be assessed by self-report measures. The similarity of a number of results, particularly of concurrent and criterion based tests of validity, in comparison with results in adults, suggest consistent patterns of relationships between the metacognitions assessed by the MCQ and mental health symptoms. Research into metacognitive theory in children and adolescents is growing; research into metacognitive therapy in this population is in its infancy but initial results are promising (Simons et al., 2006; Esbjørn et al., 2018). Further testing and development of metacognitive measures in children and adolescents should help advance this promising area of research and practice.

## Author Contributions

SM and AW were involved in study conceptualization. SM and SS were involved with systematic search, article collection and quality assessment, and data analysis. SM carried out data extraction and synthesis. All three authors contributed to the manuscript.

### Conflict of Interest Statement

The authors declare that the research was conducted in the absence of any commercial or financial relationships that could be construed as a potential conflict of interest.

## Footnotes

^1^In one study (Esbjørn et al., 2016) ranges of Cronbach alphas were given across most subscales including the CSC together rather than separately, this score is the lowest score mentioned in that study.

## Appendix

### Methodological Quality Assessment

**Research question and design**
  1                 Clear research question and appropriate design
  0                 Unclear research question and/or non-appropriate design

**Sampling method**
  1                 Appropriate for design
  0 Not appropriate

**Sample size**
  2                 Justified and satisfactory
  1                 Satisfactory, not justified
  0 Not satisfactory

**Data collection**
  2                 Validated measurement tools (of symptoms)
  1                 Non-validated tools, described and justified
  0 Non-validated tools, not described and justified

**Missing data**
  1                 Described and appropriate methods used
  0                 Not described, non-appropriate methods used

**Analysis**
  1                 Statistical tests appropriate and appropriately described
  0                 Statistical tests not appropriate and/or not appropriately described
